# Intravaginal cytomegalovirus (CMV) challenge elicits maternal viremia and results in congenital transmission in a guinea pig model

**DOI:** 10.1186/1743-422X-8-89

**Published:** 2011-03-03

**Authors:** Megan J Olejniczak, K Yeon Choi, Michael A McVoy, Xiaohong Cui, Mark R Schleiss

**Affiliations:** 1 Center for Infectious Diseases and Microbiology Translational Research, University of Minnesota Medical School, 2001 6th Street SE, Minneapolis, MN 55455, USA; 2 Virginia Commonwealth University, Department of Pediatrics, Division of Infectious Diseases, Richmond, VA 23298, USA

## Abstract

**Background:**

The objective of this study was to compare intravaginal (ivg) and subcutaneous (sc) administration of the guinea pig cytomegalovirus (GPCMV) in pregnant and non-pregnant guinea pigs. These studies tested the hypotheses that ivg infection would elicit immune responses, produce maternal viremia, and lead to vertical transmission, with an efficiency similar to the traditionally employed sc route.

**Results:**

Four groups of age- and size-matched guinea pigs were studied. Two groups were pregnant, and two groups were not pregnant. Animals received 5x10^5 ^plaque-forming units (PFU) of a GPCMV reconstituted from an infectious bacterial artificial chromosome (BAC) construct containing the full-length GPCMV genome. Seroconversion was compared by IgG ELISA, and viremia (DNAemia) was monitored by PCR. In both pregnant and non-pregnant animals, sc inoculation resulted in significantly higher serum ELISA titers than ivg inoculation at 8 and 12 weeks post-infection. Patterns of viremia (DNAemia) were similar in animals inoculated by either sc or ivg route. However, in pregnant guinea pigs, animals inoculated by both routes experienced an earlier onset of DNAemia than did non-pregnant animals. Neither the percentage of dead pups nor the percentage of GPCMV positive placentas differed by inoculation route.

**Conclusions:**

In the guinea pig model of congenital CMV infection, the ivg route is as efficient at causing congenital infection as the conventional but non-physiologic sc route. This finding could facilitate future experimental evaluation of vaccines and antiviral interventions in this highly relevant animal model.

## Background

Human cytomegalovirus (HCMV) can be transmitted by multiple body fluids, including blood, saliva, urine, breast milk, cervical and vaginal secretions, and semen. One common route by which HCMV infections are acquired is through sexual transmission. An increased number of sex partners and a history of other sexually transmitted infections both correlate with an increased risk of HCMV seropositivity [1-3]. Viral shedding has been demonstrated for protracted periods in both cervical secretions and semen [4,5]. Sexual routes of transmission in adolescent and adult patients stand in contrast to routes of transmission among children between 1-3 years of age, where salivary secretions and urine are the most common sources of HCMV [6]. What remains unclear is how the route of transmission affects parameters such as viral load, pathogenesis, immune response, and risk of vertical transmission to the fetus.

In the immunocompetent population, HCMV infection rarely results in clinical illness. In the immunocompromised and unborn, however, the consequences can be severe. HCMV remains the most common infectious cause of congenital birth defects [7]. Therefore, understanding how sexual transmission of HCMV during pregnancy affects fetal outcome is of significant concern. Among the animal models of CMV infection, the guinea-pig model has unique advantages compared to other rodent models, including establishment of infection *in utero *with resultant congenital transmission [8]. To date, virtually all studies of vertical transmission of guinea pig cytomegalovirus (GPCMV) have utilized subcutaneous (sc), intraperitoneal, or intravascular inoculation of virus, routes which may be of limited clinical relevance to acquisition of infection in humans. Therefore, these studies were undertaken to compare maternal and fetal disease following intravaginal (ivg) inoculation of GPCMV, toward the goal of developing a model of congenital transmission with greater translational relevance to human reproductive health.

A previous investigation compared immune responses to GPCMV in non-pregnant female guinea pigs following ivg, sc, or intraperitoneal inoculation [9]. This study showed that, compared to other routes of inoculation, ivg inoculation resulted in a lower percentage of inoculated animals developing detectable infection (assessed by recovery of virus in tissue culture). Vaginally infected animals also demonstrated lower titers of neutralizing antibody than animals infected by other routes. However, these studies did not examine the efficiency of infection following ivg administration in pregnant animals, in particular rates of vertical transmission to the fetus. There is also limited information about the impact of pregnancy on the natural history of GPCMV immune response and pathogenesis. In a study comparing immune responses in pregnant and non-pregnant guinea pigs, it was observed that pregnant guinea pigs developed a delayed and weaker overall antibody response following sc GPCMV inoculation compared to non-pregnant animals [10]. We therefore aimed to directly compare, using both routes of inoculation in pregnant and non-pregnant guinea pigs, the following parameters: 1) IgG response; 2) maternal DNAemia; 3) pup mortality; 4) placental viral load. In particular, we tested the hypotheses that the ivg route of infection would elicit immune responses, produce maternal viremia, and lead to vertical transmission with an efficiency similar to the more traditionally used sc route. Defining the response to ivg GPCMV administration would be of potential relevance to the study of the biology of HCMV infections, which are commonly acquired at mucosal surfaces. Further development of this model could in turn be valuable for the study of vaccines designed to elicit antibody that neutralizes virus at mucosal portals of entry and epithelial sites of primary infection.

## Results

### ELISA response to sc or ivg infection in pregnant and nonpregnant guinea pigs

**Figure 1:**
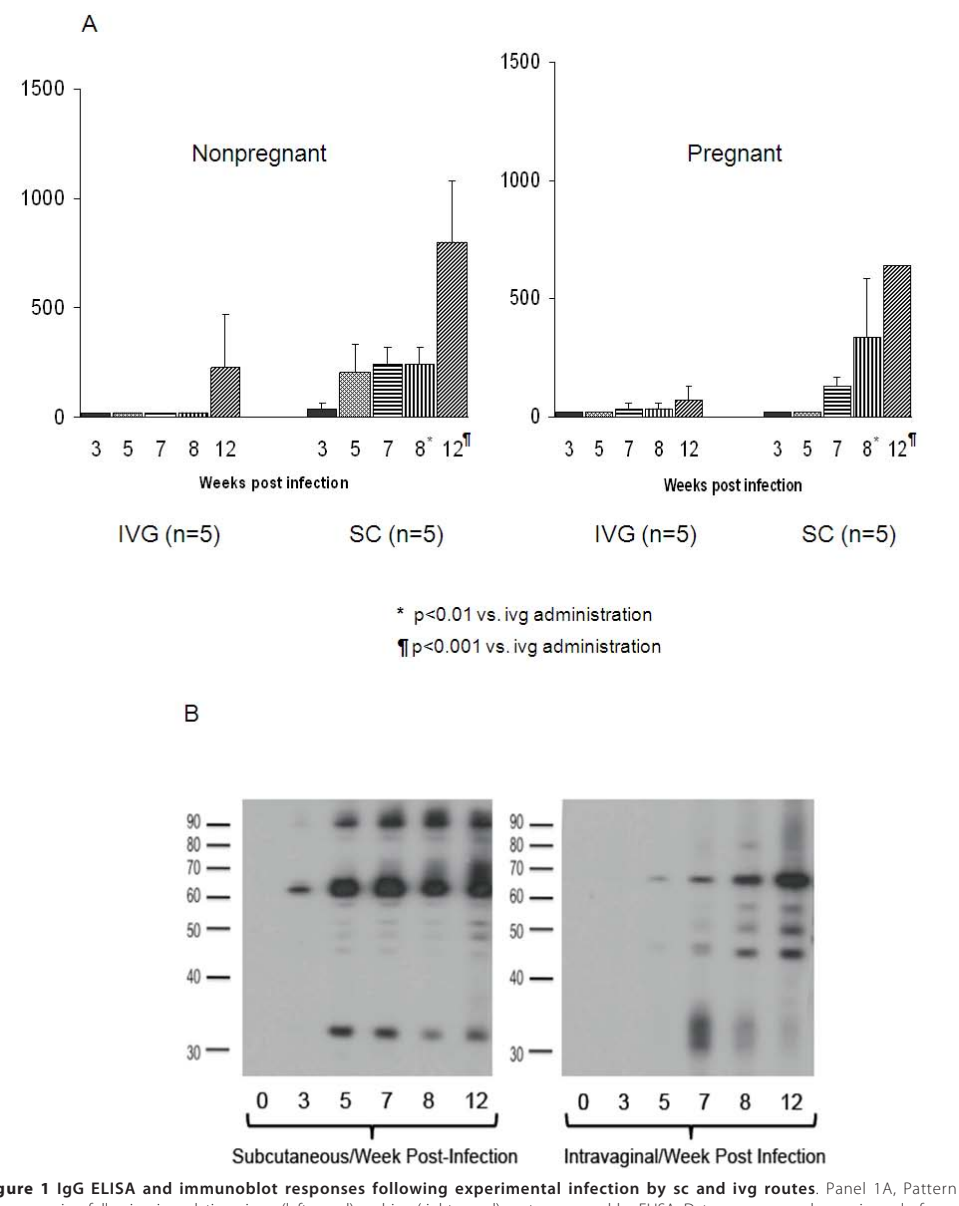
**IgG ELISA and immunoblot responses following experimental infection by sc and ivg routes**. Panel 1A, Pattern of seroconversion following inoculation via sc (left panel) and ivg (right panel) routes assessed by ELISA. Data are expressed as reciprocal of endpoint dilution titer based on limiting dilution ELISA assay as described in the text. Mean +/- SD is shown for each time point. IgG responses trended higher in non-pregnant compared to pregnant animals but no statistically significant differences were noted. Within both groups, sc inoculation elicited a higher IgG titer at week 8 post-inoculation (p < 0.01) and week 12 post-inoculation (p < 0.001) by ANOVA. Panel 1B, representative western blot analysis of temporal sequence of antibody responses in non-pregnant animal inoculated by sc route (left side of figure) and ivg route (right side of figure).

Following ivg and sc inoculation, serial serum samples were obtained for analysis of IgG response and viral load by quantitative PCR. The pattern and timing of seroconversion is shown in Figure 1. Complete data was available for 10/12 non-pregnant animals, and 10/12 pregnant animals. Statistical comparisons were performed in these animals comparing both the two routes of inoculation (sc versus ivg), irrespective of pregnancy status; and comparing pregnancy status (pregnant or non-pregnant), irrespective of the route of inoculation route.

All animals in the ivg and sc inoculation groups seroconverted to GPCMV antigen, irrespective of pregnancy status. Within each group (i.e., within the pregnant and non-pregnant groups), ELISA titers were significantly higher, at some time points, in animals infected by the sc route compared to the ivg route (Figure 1A). Antibody titer in animals inoculated by sc route was significantly higher than in animals challenged by the ivg route at both week 8 (p < 0.01) and week 12 (p < 0.001) post-infection. However, when titers were compared across groups as a function of pregnancy (i.e. comparison of pregnant to non-pregnant animals following sc inoculation, or comparison of pregnant to non-pregnant animals following ivg inoculation), no significant differences attributable to pregnancy were observed. Animals inoculated by both routes exhibited trends toward higher titers and earlier seroconversion if they were non-pregnant compared to pregnant animals, but these comparisons were not statistically significant (Figure 1A). In all infected animals, the pattern of polypeptides noted by western blot analyses after seroconversion, using GPCMV virus particles as the target antigen, was similar. However, western blot confirmed and extended the results observed by ELISA with respect to apparent differences in the temporal sequence of the antibody response, and some qualitative aspects of the response appeared different. Western blots using sera from animals inoculated by sc route demonstrated a more full repertoire of bands at earlier time points (weeks 3-7) than did the corresponding sera from animals inoculated by the ivg route (Figure 1B), although such differences were not apparent at later time points.

### Pregnancy outcomes following sc and ivg challenge with GPCMV

**Figure 2:**
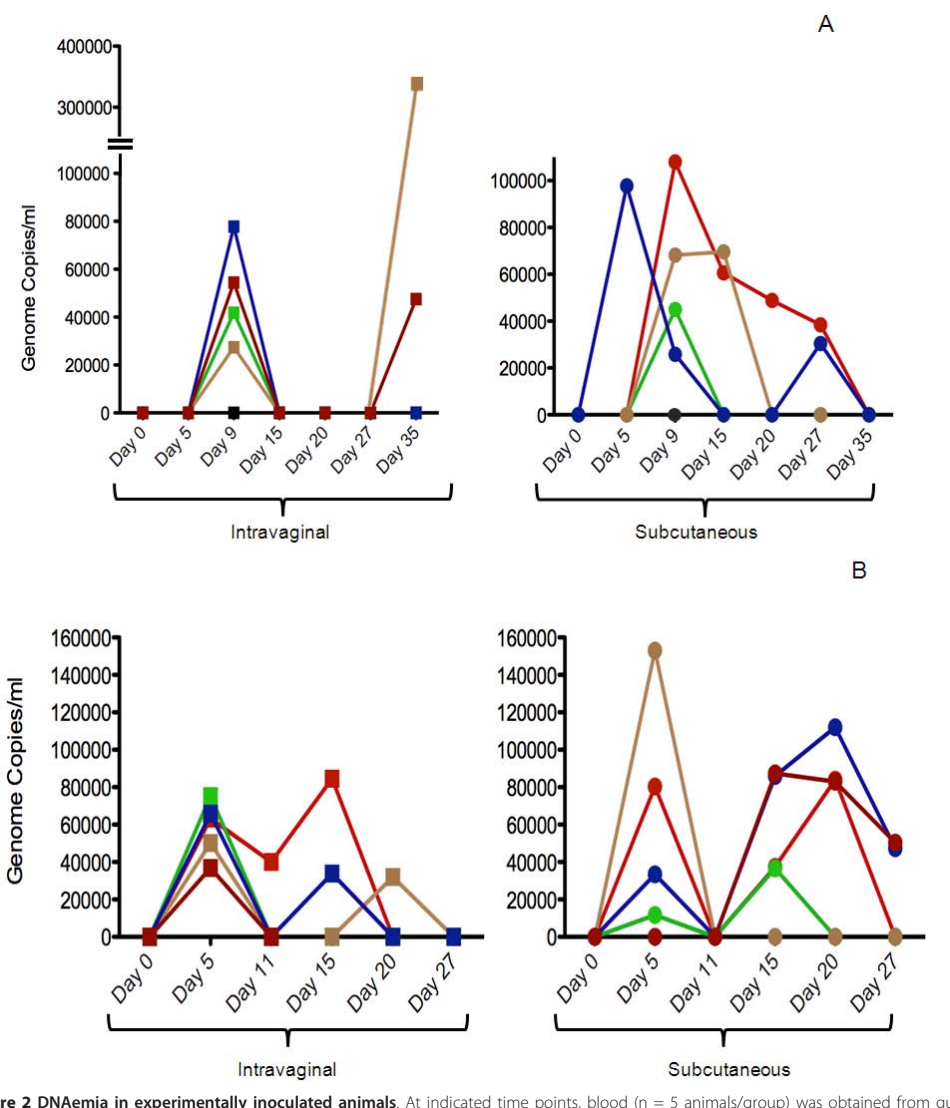
**DNAemia in experimentally inoculated animals**. At indicated time points, blood (n = 5 animals/group) was obtained from guinea pigs inoculated by ivg route (squares) or sc route (circles) and subjected to quantitative PCR for viral load determination. Each color represents an individual guinea pig. Panel 2A, profile of DNAemia following inoculation of non-pregnant animals; Panel 2B, profile of DNAemia in pregnant animals. DNAemia is noted at earlier time points (day 5 v. day 9) in pregnant animals. Biphasic pattern of DNAemia is noted in some animals.

Nearly all animals challenged by both routes demonstrated viremia (DNAemia). Of note, pregnancy was associated with an earlier onset of DNAemia, irrespective of route of challenge. Only 1/10 animals in the non-pregnant group demonstrated DNAemia at day 5 post-challenge (Figure 2A). In contrast, onset of primary DNAemia occurred within 5 days post inoculation (dpi) in 9/10 pregnant animals (Figure 2B). Although the timing of onset of DNAemia was different in pregnant animals, route of inoculation did not appear to impact the magnitude of viral load. Among DNAemic sc challenged pregnant animals, the mean systemic viral load at day 5 was 7.0 +/- 3.1 × 10^4 ^genomes/ml, whereas in ivg challenged pregnant animals, the mean viral load was 5.8 +/- 0.7 × 10^4 ^genomes/ml (P = NS). In pregnant animals, primary DNAemia had cleared by day 11 post-inoculation, but was followed by a secondary DNAemia in 4/5 sc inoculated animals; all four of these pregnant animals in this subgroup demonstrated at least one positive blood PCR between 15-27 dpi.

In non-pregnant animals (Figure 2A), primary DNAemia was observed in most animals at day 9 (8/10 animals positive), irrespective of route of challenge. DNAemia tended to be persistent in the sc challenged animals, but cleared rapidly in most ivg challenged animals; for example, in 5/5 non-pregnant ivg-infected animals, DNAemia had cleared by day 20. In two of these animals, DNAemia recurred at day 35 post-infection. No statistically significant differences were observed in the mean levels of DNAemia comparing sc and ivg challenged animals at any time point (data not shown).

### Pregnancy outcomes following sc and ivg challenge with GPCMV

Pregnancy outcomes were compared in sc and ivg inoculated dams (Table 1). Notably, pup mortality was no different between the two routes, but was significantly elevated compared to uninfected historical controls [8]. In sc inoculated animals with evaluable pregnancy outcomes (n = 5 litters), there were 15 live-born pups and 3 dead pups (mortality rate, 17%). In ivg inoculated animals with evaluable litters (n = 5 litters), there were 14 live-born pups and 3 dead pups (mortality rate, 18%; p = NS vs. sc group). Dams that delivered <7 days after viral challenge were not included in the analysis [8].

**Table 1:** Pregnancy outcomes in experimentally infected dams

Route of Inoculation	Pup Mortality	
	
	Litters	Dead/Total (%)
Subcutaneous	5	3/18 (17%)
Intravaginal	5	3/17 (18%)
Total	10	6/35 (17%)*

*p < 0.05 compared to spontaneous miscarriage rate of 4% [8]

To test whether or not ivg inoculation was as effective as sc challenge in eliciting placental and fetal infection, infection rates in live-born pups (based on analysis of pup blood samples) and in all retrievable placentas were compared using real-time PCR. In live-born pups born to the ivg challenged dams, 4/14 pups (29%) had congenital GPCMV infection (mean viral load, 5.6 +/- 2.1 × 10^4 ^genomes/ml; Table 2). In those live-born pups born to the sc challenged dams that were available for analysis, 6/10 (60%) pups had congenital GPCMV infection (p = NS compared to ivg group). The mean viral load in live-born pups born to dams in the sc inoculation group was 1.3 +/- 0.8 × 10^5 ^genomes/ml (p = NS compared to ivg group).

**Table 2:** Congenital GPCMV transmission (pup DNAemia) in live-born pups

Route of Inoculation	Pups Tested	PCR+/Total	Transmission Rate	Mean Viral Load (Genomes/ml blood)
Subcutaneous	10	6/10*	60%	1.3 +/- 0.8 × 10^5^
Intravaginal	14	4/14	29%	5.6 +/- 2.1 × 10^4^

* Only 10 of the 15 live-born pups were available for analysis

Placental infection was observed by PCR in both ivg- and sc-challenged groups. A total of 9 placentas were retrievable from this study (6 from the ivg group, from two litters, and 3 from the sc group, from two litters). It was not possible to match the placentas with individual pups. All 9 placentas were PCR positive for GPCMV DNA, with an identical mean viral load in placentas from each group (1.3 × 10^4 ^genomes/mg).

## Discussion

In this study, we compared sc and ivg inoculation of GPCMV with respect to the ability of virus to: 1) elicit immune responses; 2) establish viremia; 3) produce pup mortality and congenital GPCMV infection. We found that both routes of inoculation were able to produce viremia and cause congenital infection. To our knowledge, congenital GPCMV transmission following ivg challenge has not previously been described. In a prior report of ivg challenge in non-pregnant animals, GPCMV was noted to disseminate to salivary gland, pancreas, and lymph nodes, but pregnant animals were not examined [9]. Our study confirmed the ability of GPCMV to establish viremia following ivg inoculation in pregnant and non-pregnant animals and extends these observations by demonstrating, in pregnant animals, the transmission of virus to the placenta and fetus following this route of challenge.

We found by quantitative blood PCR that both non-pregnant and pregnant animals demonstrated DNAemia following inoculation at either sc or ivg sites. Eight of ten non-pregnant animals were DNAemic at one or more time points post-inoculation, while ten of ten pregnant animals were DNAemic. Notably, DNAemia appeared earlier in pregnant animals than non-pregnant animals after infection by either route: 9/10 pregnant animals were DNAemic at day 5 post-inoculation, compared to 1/10 in non-pregnant animals (Figure 2). This observation suggests possible differences in innate immune responses in pregnant animals compared to non-pregnant animals. DNAemia demonstrated a biphasic pattern in some animals. It is of interest to compare these observations to those previously described in a study where sc, intracardiac, and intranasal challenge were compared in pregnant animals [10]. In this study, a biphasic pattern of viremia was also observed.

In addition to maternal DNAemia, we were able to demonstrate pup mortality and congenital GPCMV infection in live-born pups delivered to dams infected by both the sc and ivg route. Pup mortality, irrespective of route of inoculation, was 17-18%, statistically significantly higher than the baseline stillbirth rate observed in guinea pigs, ~ 4% (p < 0.05, Fisher's exact test; [8]). A total of 4/14 pups (29%) born to ivg inoculated dams had congenital GPCMV infection, compared to 6/10 (60%) of pups born to sc inoculated dams. In this study, there was no discernable correlation between the timing or magnitude of maternal DNAemia and the risk of pup infection. While congenital infection rates and pup viral DNA loads both trended higher in pups born to the sc inoculated dams, these differences were not found to be statistically significant.

It was of interest to compare the IgG responses by ELISA following ivg and sc routes of infection. Animals infected by the ivg route tended to demonstrate delayed seroconversion to GPCMV, compared to the sc route, regardless of their pregnancy status. Within both the pregnant and non-pregnant groups, ELISA titers at week 8 and week 12 post-inoculation were significantly higher in animals infected by the sc route, compared to the ivg route. In the only other study that examined ivg inoculation of GPCMV, antibody titers were comparable to those following intraperitoneal or sc inoculation, but temporal patterns of antibody response were not formally compared [9]. Our data suggest that ivg infection of guinea pigs may be associated with a delayed or diminished IgG response compared to other routes of infection. If a similar delay in the antibody response to HCMV infection occurs in the setting of human pregnancy, this observation in the guinea pig model may be of relevance to pregnant women who acquire primary or recurrent infection through sexual transmission [1-4].

In this preliminary report, we chose to perform viral challenge with GPCMV reconstituted from an infectious BAC designated N13R10. The advantage of using GPCMV reconstituted from the N13R10 BAC, compared to the first generation GPCMV BAC, is that it contains a more authentic and complete genome [11]. Unfortunately, serial passage of recombinant CMVs generated in fibroblasts results in selection for genomic variants that are highly attenuated [12]. In particular, HCMV and rhesus CMV rapidly lose epithelial and endothelial cell tropisms upon serial passage in fibroblasts due to acquisition of mutations in one of three viral proteins (in HCMV UL128, UL130, and UL131) that are necessary for epithelial cell entry but dispensable for replication in fibroblasts [13-17]. An ATCC-derived GPCMV lacking homologs to the HCMV UL128-131 locus, GP129/GP131, is impaired for replication *in vivo *following sc inoculation, relative to virus that retains GP129/GP131 [18,19]. That the GPCMV generated from the N13R10 BAC retains virulence was evidenced in this study by the ability of this virus to disseminate and cause fetal transmission and disease, in contrast to the first-generation GPCMV BAC, which contains deletions, rearrangements, and mutations, and is impaired in its pathogenic potential. Subsequent to the initiation of this study, we identified that the N13R10 BAC used to generate the virus stock used for these inoculations has a 4-bp deletion/frame shift in GP129 [20]. What role, if any that this 4-bp deletion plays in modifying pathogenesis, compared to the sequence in salivary gland-passaged virus, will be addressed in future studies.

We are also in the process of determining the sequence of N13R10, as well as salivary gland-passaged GPCMV sequenced directly from tissue homogenate. This evaluation will help identify sequence variability that might play a role in pathogenesis, as well as provide information about the viral genome sequence as it exists in the context of the living animal. The FJ355434 sequence, although useful in providing a preliminary outline of the structure of the genome, has been recognized by ourselves [20] and others [21] to have limitations, due to sequence errors as well as drift from previous stocks. The FJ355434 sequence was predominately derived from overlapping plasmids constituting *Hin*d III and *EcoR *I restriction fragments and subclones repeatedly passaged over 20 years in *E. coli *[22], the only technically feasible source of template available for sequence analysis at the time those studies were conducted. Some plasmid clones dated back to the 1980 s [23] and were maintained in *E. coli *strain HB101, a *recA*13 strain in which the recombination frequency is known to be substantially greater than that observed in more contemporary *recA*1 strains [24]. It is therefore not surprising that we have noted in current studies, using deep sequencing, that there are sequence differences between the sequence derived largely from cloned plasmids; sequence from the N13R10 BACmid; and, more recently, sequence from viral DNA purified directly both from salivary gland as well as from infected fibroblasts [20]. The usefulness of N13R10 stems from the fact that it is a defined, stable reagent, which can be readily mutagenized or repaired for future *in vivo *studies. The demonstration of pathogenicity of this virus in the current study will be useful in future studies dissecting mechanisms of infection at mucosal sites of infection.

In summary, the development of a model for ivg transmission of GPCMV could have translational importance for the study of primary HCMV infection in women. In this model, we found that all retrievable placentas were infected. Since HCMV infection of the placenta [25-27] makes a major contribution to fetal injury, this observation further underscores the potential relevance of this model. These studies suggested that ivg infection with GPCMV was associated with a delayed and diminished IgG response, compared to the more traditional (but less physiologic) route of sc challenge. Also of interest, pregnant guinea pigs demonstrated earlier onset of DNAemia than non-pregnant animals, regardless of the route of inoculation. The intrinsic immunosuppression associated with pregnancy may modify the maternal response to a primary HCMV infection in the pregnant individual [28]. Strategies designed to augment innate and/or adaptive immune responses following ivg GPCMV challenge may have relevance to future interventions for prevention of HCMV infection in women and newborn infants.

## Conclusions

Ivg challenge with GPCMV can produce infection, as evidenced by an IgG response, DNAemia, and, for pregnant animals, placental and fetal infection, as well as fetal disease and mortality. The timing and magnitude of the antibody response differs between sc and ivg challenge, irrespective of the pregnancy state of the animal. DNAemia ensues earlier in pregnant animals, both in animals challenged by sc and ivg route. Finally, an infectious GPCMV reconstituted from a full-length genomic BAC cloned in *E. coli *is capable of inducing disease in guinea pigs by both routes of challenge. Further development of this model may have relevance to HCMV infection, which is commonly transmitted sexually at mucosal surfaces. Animal model studies using ivg inoculation may be of greater translational value than the more traditional but less biologically relevant sc route of challenge.

## Methods

### Animal studies

Age-matched, Hartley strain female guinea pigs (both pregnant and non-pregnant) were purchased from Harlan Industries Laboratories (Indianapolis, IN). At study outset, non-pregnant animals were 200-300 g; pregnant animals were 700-900 g, and gestation was estimated at 45 days. Animals were confirmed to be GPCMV-seronegative by ELISA prior to viral challenge. Experiments were conducted using protocols approved by the University of Minnesota Institutional Animal Care and Use Committee (IACUC). Animals were weighed weekly to monitor overall health.

### Cells and virus

Virus used in this study was derived from bacterial artificial chromosome (BAC) clone of the GPCMV genome designated N13R10 [11]. This BAC contains the full GPCMV genome. Virus was reconstituted by transfection of N13R10 BAC DNA into guinea pig lung fibroblasts (GPL; ATCC CCL-158) as previously described. Viral stocks were prepared by centrifugation and titrated by inoculating 10-fold serial dilutions on to GPL cells and counting fluorescent foci 48 hours post-inoculation [29].

### Viral challenge

A total of twelve non-pregnant guinea pigs were challenged with GPCMV (six by the sc inoculation, and six by the ivg inoculation). A total of twelve pregnant guinea pigs were also similarly challenged (six by ivg challenge, and six by the sc route). Complete data was available for 10 animals (five by each route of infection) in each group. Guinea pigs were matched for timed pregnancies at ~45 days estimated gestation (approximately at the end of the second trimester of guinea pig pregnancy). For viral infections, animals were inoculated with 5 × 10^5 ^PFU of viral stock. For ivg inoculation, animals were anesthetized using isoflurane, and inoculation was performed by inserting a lubricated polypropylene catheter into the vagina at a depth of 5 cm. The volume of the ivg inoculum did not exceed 100 μl. To ensure that minimal leakage would occur following ivg infection, anaesthetized animals were maintain in a supine position for one hour post-inoculation. Inoculation by the sc route was performed in the dorsal neck in the posterior occipital region. Following establishment of infection, blood for serology and quantitative, real-time PCR measurement of viral load was obtained by toenail clip at weekly or biweekly intervals. Blood samples were also collected from liveborn pups by toenail clip within 72 hours of delivery.

### Immune assays

Enzyme linked immunosorbent assay (ELISA) for IgG antibody was performed as previously described, utilizing GPCMV infected cell lysates as the target antigen. Lysates from uninfected cells were used as a negative control antigen [29]. For determination of antibody titers, sera were initially diluted 1:80, then titrated by additional 2-fold serial dilutions. The ELISA titer was defined as the reciprocal of the highest dilution that produced an absorbance >0.10 and was twice the absorbance observed against the control antigen. Western blots were performed on sera as described elsewhere [30].

### Viral load analysis and real-time quantitative PCR

DNA was extracted from whole blood or placental tissue extracts (10% w/v) in 15% buffered sodium citrate using a MagNA Pure^® ^LC Total Nucleic Acid Isolation Kit (Roche, Mannheim, Germany), following the manufacturer's specifications. Primers and LC probes were designed using Roche Lightcycler^® ^Probe Design 2.0 software. These primers, based on the *GP55 *(encoding glycoprotein B), were used: Forward, 5'-CTTCGTGGTTGAACGGG-3'; Reverse, 5'-GTAGTCGAAAGGACGTTGC-3'; Probe 1, 5'-TGGTGACCTTCGTTACCAATCCGTTTGGA-fluorescein; Probe 2, 5'-LC Red 640-CTTCGTGGTGTTCCTGTTCTGCGT-Phosphate. The PCR reaction mixtures were prepared using the Lightcycler^® ^Fast Start Master hybridization probes (Roche Applied Sciences) supplemented with additional 2.5 mM MgCl_2_, 0.5 μM primers and 0.2 μM probes. PCR was performed on the Lightcycler^® ^instrument using the following parameters: an initial 95°C for 10 minutes, then 45 cycles of denaturation at 95°C for 10 s, annealing at 56°C for 15 s, elongation at 72°C for 15 s. Data was collected by 'single' acquisition during the annealing step. A melting curve analysis was also performed and data acquired in the 'continuous' mode during an increase in temperature from 45°C to 85°C. Copy number was calculated based on signal ratio to control DNA as described elsewhere [31]. DNA load was expressed as total copy number/ml of blood or copy number/mg of tissue (for placental analyses).

### Statistical comparisons

Statistical comparisons were performed by ANOVA, using the InStat 3.0 software program (GraphPad Software, San Diego, CA). Following log transformation antibody and viral load data were compared across all groups. All comparisons were two-tailed.

## Competing interests

The authors declare that they have no competing interests.

## Authors' contributions

MJO conducted the experiments described in the paper. KYC developed and carried out the real-time PCR assays. MAM conceptualized the N13R10 virus, and assisted in manuscript preparation and data analysis. XC generated the N13R10 virus. MRS developed the concept for the study, analyzed data, and prepared the manuscript. All authors read and approved the manuscript.
